# Developing mentorship in a resource-limited context: a qualitative research study of the experiences and perceptions of the makerere university student and faculty mentorship programme

**DOI:** 10.1186/s12909-017-0962-8

**Published:** 2017-07-14

**Authors:** Andrew S. Ssemata, Sophia Gladding, Chandy C. John, Sarah Kiguli

**Affiliations:** 1 Department of Psychiatry, College of Health Sciences, Makerere University, Kampala, Uganda; 2 Department of Medicine, University of Minnesota, Minneapolis, MN USA; 3 Ryan White Center for Pediatric Infectious Disease and Global Health, Department of Pediatrics, Indiana University, Indianapolis, IN USA; 4 Department of Paediatrics and Child Health, College of Health Sciences, Makerere University, Kampala, Uganda

**Keywords:** Mentorship, Mentee, Mentor, Students, Faculty, Supervision, Low and middle income country

## Abstract

**Background:**

The aim of mentorship is to build the mentees capacity, enhance their skills and improve their ability to produce desired outcomes. However, the mentoring relationship is vulnerable to a number of challenges that may undermine its effectiveness and sustainability. We aimed to explore the experiences and perceptions of student and junior faculty mentees and senior faculty mentors at the Makerere University College of Health Sciences and identify the key factors defined by mentees and mentors as necessary for a successful mentorship program.

**Methods:**

A qualitative design involving focus group discussions (FGDs) and key informant interviews (KII) was used. A total of eight KII and four FGDs were conducted, audio recorded and transcribed verbatim. Open coding of the transcripts was performed, and major themes were identified through multiple readings based on thematic analysis.

**Results:**

Six key themes were shared by the mentees and mentors including: 1) defining the role of the mentor; 2) desired characteristics of a mentor and a mentoring relationship, with an emphasis on mutual trust and respect; 3) overlapping roles of mentors and supervisors; 4) issues with the process for identifying mentors, including the benefits and drawbacks of the mentee selecting mentor vs. being assigned a mentor; 5) current barriers to mentoring, including lack of knowledge about current program, lack of formal structure, uncertainly about who should initiate relationship, and unclear roles and expectations and 6) recommendations for the future development of mentoring programme, including the need for a formalized programme, and training adapted to the local context.

**Conclusions:**

The mentees and mentors described the role of the mentor and desired characteristics of mentors and a mentoring relationship similarly. Most concerns about mentoring occurred when current mentoring programmes and practices were not well aligned with these desired characteristics. Recommendations for future development of mentoring included greater formalization of mentoring with mentoring programmes based on shared expectations and adapted to the local context.

## Background

In an ideal mentoring relationship, an experienced, highly regarded, empathic person, the mentor, works with and guides another individual, the mentee, in the discovery, development and examination of their own ideas, learning and personal and professional development [1]. It is a personal and professional relationship that may continue outside school and last a lifetime with mentors taking a special interest to help their mentee reach their full potential and develop a successful career [2].

Mentoring creates opportunities for guidance, knowledge and support in a way that the mentee can willingly benefit from the mentoring relationship [3].

Mentorship in the academic health sciences has an important symbiotic relationship as the mentee is not an empty vessel receiving the mentor’s advice and wisdom but rather, an active participant, shaping the relationship [4–6]. Previous studies of mentoring in academic medicine suggest that the mentorship relationship influences academic productivity, personal development, and career guidance for students, fellows, and junior faculty [5, 7, 8].

Specifically, previous research has shown that mentors contribute significantly to the development of their mentees’ research, teaching and clinical skills, particularly with respect to career satisfaction, career management and collegial networking [4, 5, 7, 9]. The relationship benefits mentors as well, providing them with a chance to share their experience, wisdom and nurture future leaders. Therefore, mentorship should be a critical component of career and professional advancement in academic medicine.

The mentoring relationship however is prone to a number of challenges that may undermine its effectiveness and efficiency. Previous studies have indicated several challenges including: a limited pool of mentors, insufficient understanding of the mentoring process, an excessive workload for the mentor or mentee that precludes regular or in-depth mentorship meetings, and responsibilities and needs that undermine the sustainability of the mentoring programmes, e.g., clinical, research or educational demands on time [10–12].

### Mentorship at Makerere University College of health sciences

Since mentoring relationships are an invaluable way of stimulating critical thinking and are key to developing productive careers; there has been increasing and significant interest in mentorship at Makerere University College of Health Sciences (MakCHS) Uganda [10, 13]. A need to train qualified and proficient health scientists and disband the faculty ‘teach and assess knowledge’ adage has been identified [2]. Indeed, MakCHS has been a leader amongst African and other low-and middle-income countries (LMIC) in creating an institutionalized approach to mentoring [10].

Mentoring in the five clinical departments of MakCHS includes faculty-student mentorship and senior faculty-junior faculty mentorship [14]. Faculty - student mentorship involves tutoring, role modeling, clinical and research skills development and career guidance. Mentorship of junior faculty has been identified as a vital component in creating an environment that encourages faculty promotion and retention and has been credited for building indigenous research capabilities and research leadership [15, 16]. Mentoring at this level may involve but is not limited to helping in grant writing, publications and manuscript preparation, assistance in setting career targets and goals, giving constructive feedback on performance, recommending junior faculty for awards, and practice supervision [17].

It is mandatory for every post-graduate student at MakCHS to have a mentor. The mentee is assigned a mentor by their department. Each department handles the process of assigning mentors differently with mentees having no choice of mentor in any of the departments. All faculty members are eligible to be assigned as mentors with departments making decisions about who will be selected as a mentor and for how many mentees. Mentors do not currently receive any systematic training in mentoring through their departments or the college. However, there have been voluntary workshops on mentoring offered periodically. The current mentoring programming is largely unstructured beyond the assigning of mentors and the expectation that there will be one-on-mentoring between mentors and mentees throughout the mentees program of study. The mentoring of junior faculty is less well defined with junior faculty members identifying their own mentors and no formal expectations of the mentoring by the departments or college.

Most of the previous studies on mentorship have been conducted in high-income countries (HIC). In fact, all of the studies cited in two recent systematic reviews of mentoring were from HIC [5, 7]. While there are likely many similarities between mentoring in HIC and LMIC academic institutions, there may also be important differences given the importance of contextual issues in mentoring [5]. For example, differences in academic culture, particularly related to the degree of hierarchy between mentors and mentees in LMIC institutions has been previously noted as well as potential differences in incentives for mentoring and capacity for mentorship [18]. Additionally, challenges to mentorship that are experienced in both HIC and LMIC such as increasing clinical, administrative and research demands are likely exacerbated in LMIC institutions where the demands on faculty members are even greater [10, 12].

The previous research on mentoring in LMIC institutions is limited with most previous studies focusing on the mentorship of global health researchers often by HIC mentors with an emphasis on research capacity-building in LMIC [18–21]. Studies of institutional mentoring in LMIC are scarce with two recent examples from MakCHS, which have investigated the efficacy of mentoring of doctoral students [14] and a college-wide survey of mentors and mentees on current mentoring practices [10]. The experiences, challenges and needs of mentors and graduate student mentees at the clinical departmental level and the needs of faculty mentees have not been examined and the fruitfulness of a mentorship programme at this level remains unknown.

The objectives of our study were to explore and obtain a deeper understanding of the experiences and perceptions of mentoring of postgraduate students, junior faculty mentees and senior faculty mentors in the clinical departments at MakCHS specifically related to their perceptions of successful mentoring relationships, challenges and barriers to mentoring that they have experienced and recommendations for improvement. Our goal was to identify key factors needed for a successful mentorship program that would be useful not only to MakCHS but to other medical schools globally and particularly to colleges of health sciences in Africa and other low and middle income countries where institutionalized mentoring programmes have not yet taken hold [10].

## Methods

### Study setting and participants

We conducted an exploratory qualitative research involving focus group discussions (FGD) with post-graduate students (mentees) and key informant interviews (KII) with faculty members (senior faculty mentors and junior faculty mentees).

Mentees and mentors from five departments were invited to voluntarily participate in the study by email and notices placed in the postgraduate study rooms and departmental notice boards. The five departments were purposefully chosen to provide variability in number of mentees (including departments with many and departments with few students) and availability of faculty to participate in the study. We purposively recruited mentees from all 3 years of training and mentors with a range of years of experience in order elicit multiple perspectives on the mentoring programme.

We present data of twenty-three postgraduate student mentees including eleven from Internal Medicine, six from Paediatrics and Child Health, and six from Psychiatry. Of the 23 mentees, 6 (26%) were first year students and 17 (74%) were second or third year students. Eight faculty members agreed to participate in the study including two each from Paediatrics and Child Health, Psychiatry and Surgery, one from Obstetrics and Gynecology and one senior administrator from the central administration of MakCHS.

All participants were aware of the mentoring programme at MakCHS and had at least some involvement with the programme. In general, the first year mentees had the least experience having been students for 8 months at MakCHS when the study was held. All first year mentees had at least made initial contact with their mentors. The second and third year mentees had more experience in the mentoring programme having had more interaction with their mentors though the amount of interaction varied. For the faculty members, all were current mentors in the mentoring programme. The senior administrator is also a senior faculty member and has been a mentor in the program as well as being involved in the administration of the mentoring programme.

### Study design

A qualitative research design, which is an interpretative method of naturalistic inquiry with a focus on exploring, describing, and interpreting socio - personal experiences and meanings participants attach to their social world represented in language [22, 23] was adopted.

### Data collection

Five focus group discussions (2 FGD in Internal Medicine, 1 in Paediatrics, 1 in Psychiatry, 1 in Obstetrics and Gynecology) and eight key informant interviews were conducted from May – July 2013. We present data from four FGDs as the audio recording from the Obstetrics/Gynecology group was almost inaudible, therefore no accurate transcription could be done. All interviews were conducted in English (the language of instruction at MakCHS) by an experienced interviewer (AS) using a KII schedule and FGD guide (Table 1).

**Table 1:** Focus group and key informant interview questions regarding mentees and mentors experiences and perceptions of the mentorship programme at the School of Medicine, College of Health Sciences, Makerere University

Focus group questions to mentees: • What is mentoring to you? ◦ How would you define mentoring? ◦ What elements constitute mentoring? • Tell me about your mentorship relationships. How do you feel about the meetings you have? ◦ How did you get your mentor? ◦ How often do you meet? • Mentoring relationships are supposed to be/expected to be reciprocal. How do you feel this has been met in your relationship? ◦ Did you have a mentor at undergraduate and how is it different from your current relationship as a post-graduate student? • Think about the last meeting with your mentor, what did you like or not like about your meeting? ◦ How did you feel at the end of the meeting? • In your view, what would consider as an ideal mentorship relationship? ◦ What to expect? What would you like to see or be done? • Is there something about the current mentorship programme that should be upheld, enhanced, maintained? • What would you like to see change about your mentorship relationship? ◦ and mentorship at the college? • Do you have anything you would like to share concerning mentorship at the college that we have not discussed or talked about?
Key informant interview questions to mentors: • What is mentoring to you? ◦ How would you define mentoring? ◦ What elements constitute mentoring? • Tell me about your mentorship programme or initiatives in the department? ◦ Who is involved? How are they involved? • How is the mentoring programme being conducted? ◦ Who assigns the mentors ◦ How many mentees per mentor) • Specifically, is there faculty mentorship in the department and how is it being conducted? • Are you involved in mentoring or are you being mentored? ◦ What is your personal experience? • In your view, what would you consider as an ideal mentorship relationship? ◦ What to expect? What would you like to see or be done? ◦ Is it any different from how you were mentored or are being mentored? • Did you have a mentor as a faculty and how is it different mentoring junior faculty? ◦ Tell me what you found different • Think about the last meeting with your mentor, what did you like or not like about your meeting? ◦ How did you feel at the end of the meeting? • What would you like to see change about mentorship at the department? ◦ and mentorship at the college? • Do you have anything you would like to share concerning mentorship at the college that we have not discussed or talked about?

The interviewer was not known to the FDG participants and was known to only one of the KII participant prior to the interviews. The interviews lasted 50–80 min and were conducted in a neutral place for both the researcher and participants to encourage balance of power. All participants were debriefed at the end of each discussion group and interview session and a debrief form was provided.

### Data analysis

The discussions were audio recorded and transcribed verbatim and the transcripts analyzed along side the audio recording to ensure accurate transcriptions such that meaning was not lost. We then conducted a thematic analysis of the transcripts [24] using two authors (AS and SG) to provide investigator triangulation in which multiple investigators are used to provide corroborating evidence [25]. Two authors, one who conducted the interviews and one who has no involvement with the mentoring programme independently read each transcript multiple times and coded the transcripts using open coding [26]. After the initial coding, the two authors compared their codes and created a shared set of final codes. The same two authors then coded the transcripts again using the agreed upon set of codes, after which they compared their coding for each transcript, discussing areas of disagreement until consensus was reached [27]. The two authors then reviewed the coded text to identify themes by looking for patterns of connection and clustering related codes into broader categories [23]. Transcripts for the FGDs and KIIs were considered separately as we were interested in examining similarities and differences between mentees and mentors, however, after coding the transcripts and reviewing the coded text when creating the over-arching themes, it became apparent that the themes were consistent across the FGD and KII transcripts though there were distinct codes for the FGDs and KIIs that supported the themes. The research received ethical clearance and approval from by School of Medicine Research Ethics Committee and Uganda National Council for Science and Technology.

## Results

We identified six key themes including: 1) defining the role of the mentor; 2) desired characteristics of a mentor and the mentoring relationship; 3) the overlapping roles of mentor and supervisor; 4) the process for identifying mentors; 5) current barriers to effective mentoring, and 6) recommendations for the future development of mentoring programmes (Table 2).

**Table 2:** Themes from focus group discussions and key informant interviews with mentees and mentors regarding their experiences and perceptions of the mentorship programme at the School of Medicine, College of Health Sciences, Makerere University

Theme	Mentees perceptions	Mentors perceptions
The role of the mentor	• Provides career, academic and personal guidance • Provides motivation and encouragement to mentee • Help mentees realize their strengths and minimize weaknesses	• Provides career, academic and personal guidance • Acts as a role model • Provides guidance regarding professionalism and ethical issues
Desired characteristics of a mentor and mentoring relationship	• Mutual trust and respect • Shared interests • Based on established relationship	• Mutual trust and respect • Shared interests • Relationship free of intimidation with mentee feeling empowered
Overlapping roles of mentors and supervisors	• Can lead to conflicts of interest • Can lead to lack of trust to share non-academic issues • Potential benefit of shared interest and investment in project	• Can lead to conflicts of interest
Issues of identifying mentors	• Benefits of mentee selecting mentor leading to more successful, authentic and reciprocal mentoring relationship • Drawbacks of mentee selecting mentor include mentors having too many mentees and mentees may have difficulty identifying a mentor • Drawback of assigned mentor is that mentees may not be comfortable with the relationship	• Benefits of mentee selecting mentor leading to more successful, authentic and reciprocal mentoring relationship • Drawbacks of mentee selecting mentor include mentors having too many mentees and mentees may have difficulty identifying a mentor • Assigned mentoring relationships may be difficult to sustain
Barriers to mentoring	• Lack of knowledge about current mentoring programme • Lack of formal structure • Uncertainty about who should initiate relationship • Unclear roles and expectations • Lack of mentoring skills	• Lack of knowledge about current mentoring programme • Lack of formal structure • Uncertainty about who should initiate relationship • Unclear roles and expectations • Lack of designated time for mentoring • Lack of documentation, feedback and accountability in mentoring
Recommendations for future development of mentoring program	• Need for more formalized programme • Create shared mentor/mentee understanding and expectations of mentoring • Need for mentoring model adapted to local context	• Need for more formalized programme ◦ College requirements for number of mentoring meetings ◦ Assistance with scheduling mentoring meetings ◦ Measurable outcomes for mentoring ◦ Recognition for mentoring ◦ Place where concerns about mentoring can be taken ◦ Create shared mentor/mentee understanding and expectations of mentoring • Need for mentoring model adapted to local context

### Defining the role of the mentor

The mentees and mentors provided similar definitions for the role of the mentor. They described the important roles of the mentor as including: providing career, academic and personal guidance; providing motivation and encouragement to the mentee as well as helping mentees realize their strengths and minimize their weaknesses. Mentors emphasized the role of the mentor as a role model and particularly providing guidance in professionalism and ethical issues. In terms of mentorship of junior faculty, mentors indicated that the important role of the mentor is to share opportunities and provide support and guidance in promotion.

### Desired characteristics of a mentor and a mentoring relationship, with an emphasis on mutual trust and respect

The mentees and mentors also provided similar descriptions of the desired characteristics of a mentor and of a mentoring relationship. Both mentors and mentees described the importance of mutual trust and respect. They indicated that the mentoring relationship was most likely to be successful if it was reciprocal and based on shared interests and an established relationship.

*“I think on the whole the mentor- mentee relationship is most enjoyed when you have an existing relationship even if it’s a prior relationship and the person knows you, you have some bit of trust.” Mentee*

Mentees and mentors also shared that to be successful the mentoring relationship needs to be a comfortable one in which the mentee could share personal issues. The mentors emphasized that in order for this to happen the mentoring relationship needs to be free of intimidation and the mentee needs to feel empowered.

### Overlapping roles of mentors and supervisors

Mentors reported that there is often an overlap in their role as a mentor and a research supervisor.

*“If you are supervising a student, you are also mentoring them. The distinction is often blurred for post-graduate student…so it could be that you are a supervisor but also a mentor, there is* mentorship beyond supervision.” *Mentor*

This overlap in roles raised some concerns for both mentors and mentees namely that if a faculty member is a student’s supervisor and mentor, there can be a conflict of interest.

*“I found that… if I had problems with my supervisor, it was easy for me to talk to my mentor, but now if your mentor is your supervisor and you are having problems with the supervisory part and then whom are you going to talk to? And you find you’re stuck.” Mentor*

Mentees acknowledged that there could be benefits to having their supervisors also be their mentors as it guarantees shared interests and investment on the part of the faculty member.

*“I meant that they [research projects] give them [student and research supervisor] something to do together, and I think the research project is one of a kind because, it involves a lot of input, and that’s where the mentor should invest in their mentee…and they should be able to meet regularly and frequently rather than meeting twice in three years*” *Mentee*

Mentees however, also raised some concerns that having the same person serve as mentor and supervisor, specifically, that they may cause a lack of trust to share non-academic issues.

### Issues with identifying mentors, including the benefits and drawbacks of the mentee selecting mentor versus being assigned a mentor

Both the mentees and mentors identified the process for identifying mentors as a critical consideration in mentoring. They both indicated that mentoring relationships were more likely to be successful if the mentee selected their mentor. Both groups described that when mentees choose their mentors, the relationships are more likely to be authentic, more reciprocal and based on shared interests.

*“Where the mentee has had to select a mentor, there will be a sense of responsibility for both parties… you have to have a level of relationship that can help you share freely so that you are not only saying… [you are] working hard and everything is fine,… but you want to bring out the other issues that are affecting you and will help you do the course better and focus you to the life after as well” Mentee*

Both groups expressed some logistical concerns if the mentees selected their mentors. In particular, mentees expressed concern that some mentors may have too many mentees and that post-graduate students who did not have established relationships with faculty members might find it difficult to identify a mentor. The mentors shared similar concerns particularly regarding the need to have an even distribution of mentees across mentors.

If mentees are assigned to mentors, both mentees and mentors expressed limitations of this model. Mentees described how if the mentor is assigned it may limit the mentoring relationship.

*“We are generally scared, you know you are given a mentor and they are your assessor and examiner, you cannot open up completely because if they know that this man was not ready for this programme, it could bias their way of thinking when they are assessing you. The mentorship then becomes like a movie. You are there, you have to sieve and speak the right words.” Mentee*

Mentors raised the issue that if mentors are assigned, the mentoring relationship may be difficult to sustain.

*“And with the postgraduates…so, many of them come in first year and they are assigned mentors…its usually difficult to follow through…so by 2* ^*nd*^ *year or 3* ^*rd*^ *year, the kind of the relationship which may have begun kind of dies out.” Mentor*

### Current barriers to mentoring, including lack of knowledge about current programme, lack of formal structure, uncertainly about who should initiate relationship, and unclear roles and expectations

Both mentees and mentors reported a number of barriers to effective mentorship. Both agreed that there is currently a lack of a formal mentoring structure; those in assigned mentorship relationship may not know each other or have ever interacted; there is uncertainty about who should initiate the relationship; there is a lack of clarity around roles and expectations, and a concern that mentoring only occurs when there is a problem.

*“The mentor they assigned to me, I really didn’t know the guy and we would even pass each other in the corridors, he didn’t know who I was and so it became difficult. It was going to be a long time to build the relationship and friendship.” Mentee*

The mentees additionally described that there is a lack of knowledge about the existing mentoring program and a lack of mentoring skills. Mentors identified a lack of designated time and commitment to the relationship, lack of documentation, feedback and accountability in mentorship as additional barriers.

*“The mentors are often very busy and they have other activities and they don’t have the time to do the mentoring very well and the mentees are usually busy but they are also scared so they also try to find an excuse of not going, so that’s the challenge.” Mentor*

### Recommendations for the future development of mentoring programme, including the need for a formalized programme, and training adapted to local context

Participants identified multiple recommendations for the future development of the mentoring programme at MakCHS. The recommendations included: the need for a more formalized mentoring programme, the need for mentor/mentee training to create a shared understanding and expectations for mentoring, and the need for a mentoring model adapted to the local context.

#### Need for a more formalized program

Both mentees and mentors identified a need for a more formalized and structured mentoring programme. Mentors suggested that this could include the College requiring a certain number of mentoring meetings between mentors and mentees, assistance with scheduling the initial mentoring meeting, the need for a measurable outcome for mentoring, recognition for the additional effort of mentoring and a coordinating place where mentors and mentees can go with concerns about mentoring.

*“Mentorship needs to be formalized, because … most of us are so busy and I think if it is not formalized then it doesn’t happen” Mentor*

#### Need for mentor/mentee training to create a shared understanding and expectations for mentoring

Participants identified a need for training to create a shared understanding of mentoring between mentees and mentors including shared expectations, roles and responsibilities.

*“We need to be trained; both the students and the lecturers. There is need for formal training after assigning if it is important to the college. When the first years come, they should be trained on mentorship, the importance of mentorship.” Mentee*

#### Need for a mentoring model adapted to the local context

Participants suggested the college should design a mentoring model adapted to the local context. There were discussions about the existing divide in relation to respect, hierarchy and levels of authority at the college and its impact on the mentoring relationships. Additionally, for junior faculty mentorship, individualism and competition were reported to affect faculty mentoring at the departments.

*“I think the Ugandan culture has a lot of hierarchy, adults-children, seniors-juniors, first years-third years; so this mentoring thing is not yet natural so we have to find a way to incorporate it in our society. In other societies the relationship with your professor can be more informal and you find that you can freely interact with the senior with due respect. We may have borrowed this mentorship thing from elsewhere but we have not formalized how to make it Ugandan.” Mentor*

In addition to developing a mentoring model adapted to the local context culture, it was also suggested that MakCHS must develop its own definition of mentoring.

*“I think Makerere and the College of Health Sciences… has to define what is the mentorship that they want, we have discussed different forms, we have not yet defined what is it that we want … and we follow that given the different types, given the realities that we want and given the different levels of training that we have.” Mentor*

## Discussion

Participants in our study indicated the importance of mutual trust and respect in mentoring relationships and that the mentoring relationship is most likely to be successful if it is reciprocal and based on shared interests. This description is similar to the results of a previous study of mentoring at two North American academic health centers who found that the characteristics of successful mentoring relationships included reciprocity, mutual respect, clear expectations and shared values and interests [11]. Additionally, our results mainly related to the personal characteristics as compared to other studies, which have identified desired characteristics of mentors in the relational and professional dimensions [7]. This suggests that the personal dimension may be of particular importance in contexts where there is a high degree of hierarchy between mentors and mentees with mentors and mentees both emphasizing the importance of mutual trust and respect.

Our findings further suggest that concerns about mentoring mostly occur when participants’ experiences with the current mentoring programmes and practices are not well aligned with the desired characteristics of successful mentoring relationships. Specifically, mentors and mentees raised concerns about the overlap in roles between mentors and research supervisors noting that when a faculty member serves as both a mentor and research supervisor, the mentoring relationship may no longer be based on mutual trust. This creates concerns including potential conflicts of interest and reluctance from students to share their problems and non-academic issues. These concerns with the overlapping roles have been documented in previous studies [8, 9]. However, in these studies, the overlap was between supervisors and informal mentors. The overlapping roles between a supervisor and a formal, assigned mentor may be even more difficult as there are expectations for both relationships that may be in conflict. This situation of overlapping roles is likely more common in resource-constrained institutions where there are fewer faculty members who must fulfill multiple roles.

Mentees and mentors also expressed concerns related to the process for identifying mentors and whether mentors are assigned or selected. If mentors are assigned, mentees and mentors indicated that the mentoring relationship was less likely to be based on mutual respect and shared interests and, therefore, less likely to be successful. This concern again seems to reflect a lack of alignment between the practice of assigning mentors and the desired characteristics of mentoring relationship based on mutual respect and shared interests. Indeed mentees and mentors shared that the current practice of assigning mentors was a barrier to mentoring, with mentors and mentees less likely to meet or interact if they do not know each other. Previous research on assigned or self-identified mentors has identified this as an important area that needs further research [5]. One study of a mentee-identified mentoring programme for junior faculty found lack of chemistry between mentors and mentees [4] to be a barrier to mentoring while Jackson and colleagues [13] found that successful relationships could happen informally, through relationships that evolve naturally over time or from formal assigned mentoring relationships, suggesting that assigning mentors is not always a barrier and underscoring the need for more research in this area.

To address these concerns and the current barriers to mentoring, the recommendations to improve the mentoring programme at MakCHS suggested by mentees and mentors involved bringing the current mentoring programmes and practices into better alignment with their descriptions of the desired characteristics of mentors and mentoring relationships. Importantly, it was also expressed that the mentoring programme should be designed for the local context, reflecting the local college- specific definition of mentoring and desired characteristics of mentors and mentoring. Designing a mentoring programme specifically for the local context would maximize the alignment with the local definitions and desired characteristics of mentors and mentoring and the likelihood of success of a mentoring programme. This recognition of role of local context is consistent with previous research that has emphasized the importance of contextual issues in mentoring [5].

Additionally, the mentees and mentors indicated a need for a more formalized mentoring programme to ensure access to and accountability in the mentoring process. In particular, mentors suggested the need for greater infrastructure to support growth in mentoring as well as more formal acknowledgement of the role of mentoring. These recommendations likely reflect the many demands on both mentees and mentors that are particularly acute in low-resource settings. These suggest that additional support will be needed to develop and sustain a robust mentoring programme.

Our study has several limitations. While we involved post-graduate students and faculty members from each of the departments included in our study, the number of participants was not uniform across focus group discussions or departments. Additionally, we offered open invitations to participate in the research and, therefore, we included participants who had limited experience of mentoring. Consequently, we may not have captured the full range of perspectives and opinions within the college regarding mentoring.

## Conclusions

Mentorship at MakCHS was considered important by both mentors and mentees with most concerns reflecting a lack of alignment between the current mentoring programmes and practices and their definitions of desired characteristics of mentors and mentoring relationships. Important next steps include; a) the design and establishment of a formal mentorship programme for the graduate students and faculty; b) developing mentoring metrics through which the progress of the mentorship programme can be measured; c) longitudinal studies of mentees to determine the longer-term impacts of mentorship. Mentorship is critical, as the post-graduates will become future mentors and essential to the future success of the mentorship programme.

## Abbreviations

FGD: Focus Group Discussion
KII: Key informant interview
LMIC: Low and middle income country
MakCHS: Makerere University, College of Health Sciences

## References

[1] Standing Committee on Postgraduate Medical and Dental Education (1998). Supporting doctors and dentists at work: an inquiry into mentoring.

[2] Mugyenyi P, Sewankambo N (2010). Mentors' manual for health sciences training in Africa.

[3] Feldman M (2012). Faculty mentoring toolkit: UCSF faculty mentoring program.

[4] Pololi L, Knight S (2005). Mentoring faculty in academic medicine. JGIM.

[5] Sambunjak D, Straus SE, Marušić A (2006). Mentoring in academic medicine: a systematic review. JAMA.

[6] Zerzan JT, Hess R, Schur E, Phillips RS, Rigotti N (2009). Making the most of mentors: a guide for mentees. Acad Med.

[7] Sambunjak D, Straus SE, Marusic A (2010). A systematic review of qualitative research on the meaning and characteristics of mentoring in academic medicine. JGIM.

[8] Rose GL, Rukstalis MR, Schuckit MA (2005). Informal mentoring between faculty and medical students. Acad Med.

[9] Leslie K, Lingard L, Whyte S (2005). Junior faculty experiences with informal mentoring. Med Teach.

[10] Nakanjako D, Byakika-Kibwika P, Kintu K, et al. Mentorship needs at academic institutions in resource-limited settings: a survey at Makerere University College of health sciences. BMC Med Educ 2011;11(1):1. 10.1186/1472-6920-11-53 PMC3170866 21801406

[11] Straus SE, Johnson MO, Marquez C, Feldman MD (2013). Characteristics of successful and failed mentoring relationships: a qualitative study across two academic health centers. Acad Med.

[12] DeAngelis CD (2004). Professors not professing. JAMA.

[13] Jackson VA, Palepu A, Szalacha L, Caswell C, Carr PL, Inui T (2003). “Having the right chemistry”: a qualitative study of mentoring in academic medicine. Acad Med.

[14] Nakanjako D, Katamba A, Kaye DK (2014). Doctoral training in Uganda: evaluation of mentoring best practices at Makerere university college of health sciences. BMC Med Educ.

[15] Marie Block L, Claffey C, Korow MK, McCaffrey R (2005). The value of mentorship within nursing organizations. Paper presented at: nursing forum.

[16] Leners DW, Wilson VW, Connor P, Fenton J (2006). Mentorship: increasing retention probabilities. J Nurs Manag.

[17] Palepu A, Friedman RH, Barnett RC (1998). Junior faculty members' mentoring relationships and their professional development in US medical schools. Acad Med.

[18] Bennett S, Paina L, Ssengooba F, Waswa D, M’Imunya JM (2013). Mentorship in African health research training programs: an exploratory study of Fogarty International Center programs in Kenya and Uganda. Educ Health.

[19] Manabe YC, Katabira E, Brough RL, Coutinho AG, Sewankambo N, Merry C (2011). Developing independent investigators for clinical research relevant for Africa. Health Res Policy Syst.

[20] Heimburger DC, Carothers CL, Gardner P, Primack A, Warner TL, Vermund SH (2011). Nurturing the global workforce in clinical research: the National Institutes of Health Fogarty international clinical scholars and fellows program. Am J Trop Med Hyg.

[21] Whitworth JA, Kokwaro G, Kinyanjui S (2008). Strengthening capacity for health research in Africa. Lancet.

[22] Smith JA (2007). Qualitative psychology: a practical guide to research methods.

[23] Bowling A (2014). Research methods in health: investigating health and health services.

[24] Boyatzis RE: Transforming Qualitative Information. Thematic Analysis and Code Development. SAGE Publications Inc.; 1998. http://www.sagepub.com/books/Book7714. Accessed 24 Oct 2015.

[25] Lincoln YS, Guba EG (1985). Naturalistic inquiry.

[26] Creswell JW (1999). Qualitative inquiry and research design: choosing among five designs.

[27] Denzin NK, Lincoln YS (1998). Collecting and interpreting qualitative materials.

